# Optimal Tracking Control of a Nonlinear Multiagent System Using Q-Learning via Event-Triggered Reinforcement Learning

**DOI:** 10.3390/e25020299

**Published:** 2023-02-05

**Authors:** Ziwei Wang, Xin Wang, Yijie Tang, Ying Liu, Jun Hu

**Affiliations:** College of Electronic and Information Engineering, Southwest University, Chongqing 400700, China

**Keywords:** neural networks (NNs), optimal tracking control, event-triggered mechanism, reinforcement learning (RL), systems with multiple agents

## Abstract

This article offers an optimal control tracking method using an event-triggered technique and the internal reinforcement Q-learning (IrQL) algorithm to address the tracking control issue of unknown nonlinear systems with multiple agents (MASs). Relying on the internal reinforcement reward (IRR) formula, a Q-learning function is calculated, and then the iteration IRQL method is developed. In contrast to mechanisms triggered by time, an event-triggered algorithm reduces the rate of transmission and computational load, since the controller may only be upgraded when the predetermined triggering circumstances are met. In addition, in order to implement the suggested system, a neutral reinforce-critic-actor (RCA) network structure is created that may assess the indices of performance and online learning of the event-triggering mechanism. This strategy is intended to be data-driven without having in-depth knowledge of system dynamics. We must develop the event-triggered weight tuning rule, which only modifies the parameters of the actor neutral network (ANN) in response to triggering cases. In addition, a Lyapunov-based convergence study of the reinforce-critic-actor neutral network (NN) is presented. Lastly, an example demonstrates the accessibility and efficiency of the suggested approach.

## 1. Introduction

Recently, distributed coordination control of MASs has received a great deal of attention as a result of its extensive applications in power systems [1,2], multi-vehicle [3] and multi-area power systems [4], and other fields. MASs have a variety problems, such as consensus control [5,6,7], synchronization control [8,9], anti-synchronization control [10], and tracking control [11]. Reinforcement learning (RL) [12] and adaptive dynamic programming (ADP) methods [13,14] have been employed by researchers as a means of solving the optimal control problems. Due to its excellent ability for global approximation, neural networks are excellent for dealing with nonlinearities and uncertainties [15]. ADP has great online learning and adaptive ability when it uses neural networks. Furthermore, researchers used RL/ADP algorithms to settle optimal coordination control matters, proposed a lot of directions, tracked control [16,17,18,19], graphical games [19], consensus control [20], containment control [21] and formation control [22]. The controller is designed in the above ways were relying on traditional time-triggered methods. Event-triggered in [23,24], it was suggested that the traditional implementation be changed to an event-triggered one. Because of the increasing number of agents, MASs are required to resolve many computing costs related to the exchange of information. Traditionally, the controller or actuator is constantly updated over a fixed period while the system is in operation. In order to minimize computation and preserve resources, aperiodic sampling is employed in the method of triggering events to improve the controller’s computation efficiency. There have been a number of developments in methods that are based on events for addressing discrete time systems [24]. The traditional implementation was suggested to be replaced by one that is triggered by events.

With an increase in the number of agents, MASs must solve a large number of computing costs related to information exchange. Traditionally, the controller or actuator is constantly updated frequently using a predetermined period of sampling during system operation. To lessen the computational and save resources, aperiodic sampling is used in the event-triggering scheme to improve the associated controller’s computational efficiency. Researchers have developed some event-based methods to address discrete time systems [25] as well as systems based on continuous time [26,27]. Several algorithms based on triggered events have been designed to solve discrete-time systems [25], as well as systems that operate in continuous time [26,27]. According to these results, the system dynamics are assumed to be accurate ahead of time. However, it is not always possible to understand dynamics properly in practice. According to [24], a controller that was triggered by events was proposed which was designed with inaccurate or unknown dynamics for the system.

The application of Q-learning to process control [28], chemical process control, industrial process automatic control, and other areas was an early application of reinforcement learning (RL). The Q-learning algorithm provides a modeless data-driven method for solving control problems. A key point to keep in mind is all potential actions in the present state. Q-learning is currently used primarily for routing optimization and reception processing in network communication within the context of network management. The Q-learning algorithm supports a modeless data-driven method for solving control problems. It is important to note that all potential actions in the present state [29] are evaluated in the Q-learning method, relying on the Q-function. At present, Q-learning is used primarily for routing optimization and reception processing in network communication in the domain of network management [30]. As a result of AlphaGo’s emergence, dynamic research has been conducted in the field of game theory, and tracking control research has been conducted on issues associated with nonlinear MAS tracking control based on Q-learning, such as in [31]. At present, there is some research for tracking control issues for nonlinear MASs based on Q-learning, such as in [32].

The MAS’s issue of optimal control was solved using the RL/ADP method, as mentioned above. The majority of the above results share two common features. First, the direct use of the immediate or immediate reward (IR) signal to define each agent’s performance index function results in limited learning opportunities. As a second step, a state’s value function is used to determine the Hamilton–Jacobi–Bellman (HJB) equation. The corresponding controller is designed using RL/ADP, which results in efficient learning of the MAS equation. It is beneficial to provide each agent with more information signals in a wide range of realistic applications in order to enhance their learning capabilities. In addition to merely considering performance in terms of status, performance can also be viewed from a broader perspective. The purpose of our research is to avoid the limitations described above.

Taking into consideration the aforementioned findings, this work investigates an ideal solution to the optimum control issue for MASs with unknown nonlinearity to enhance the process of learning as well as the effectiveness of control systems. Utilizing the graph theory, a coordination control problem is first identified. According to the gathered information of the IR, increased reinforcement reward (IRR) signals are provided for a longer-term reward period. Based on the IRR function, a Q-function is then developed to assess the efficacy of each agent’s control system. In addition, a tracking control technique is developed using iterative IrQL to derive the HJB equation for each agent. Then, based on the IrQL technique, triggering mechanisms are employed to establish a tracking control system. Finally, an optimum event-triggered controller based on a network topology of reinforce-actor-critic is created. The event triggering mechanism in a closed-loop approach guarantees that the network weights converge and the system remains stable. In light of the findings of this study, an additional contribution has been made to the literature:

(1) With respect to nonlinear MAS tracking control, the authors of [32] proposed an IrQL framework, which differs from [18,33,34], and the design of a new long-term IRR signal is completed. This product was designed on the basis of the data of neighbors to provide more information to the agent. The IRR function is used to define a Q-function, and an iterative IrQL method is proposed for obtaining control schemes that are optimally distributed.

(2) It is designed to trigger a new condition and cite in an asynchronous and distributed manner [24]. As a result, each agent triggers at its own time. Consequently, there is no need to update the controller on a regular basis. For the purpose of achieving online learning, a reinforce-actor-critic neural network based on triggered events is established to determine the optimal control scheme for triggered events. When compared with other papers [18,33,35,36], this paper adjusts the weights non-periodically, and the ANN is only adjusted when a trigger is encountered.

(3) In this paper, the objective is to develop the most effective tracking control method using a new triggering mechanism developed using the IrQL method. As far as event-triggered optimal control mechanisms are concerned, the Lyapunov approach is used to determine the rigorous stability assurance of closed-loop multi-agent networks. The designed RCA-NN framework [32] offers an effective means of executing the proposed method online without requiring any knowledge of the dynamics of the system. We made a comparison between the traditional activation method and the IrQL method. According to the simulation results, the designed algorithm is capable of detecting control problems with good tracking performance.

This article is organized as follows. The graph theory and problems of Section 2 provide an overview of some foundations. In Section 3, IrQL-based HJB equations are obtained. As described in Section 4, the most appropriate controller design should be triggered by an event to build the proposed algorithm. Section 5 develops the RCA-NN. The use of Lyapunov technology leads to convergence of weights in the neural networks. Through analogy examples and comparisons, its effectiveness and correctness of the method are demonstrated in Section 6. The last part includes our final thoughts.

## 2. Preliminary Findings

### 2.1. Theoretical Basis of Graphs

It would be possible to model the exchange of information using a directed graph between agents G=(V,E,A), in which $V=\left\{\upsilon_{1},\upsilon_{2},\ldots ,\upsilon_{n}\right\}$ represents N nonempty notes and $E=\{\left(\upsilon_{i},\upsilon_{j}\right)|\upsilon_{i},\upsilon_{j}\in V\}\in V\times V$ represents an edge set, indicating agent *i* could derive the data from agent *j*. We define $A=[a_{ij}]$, which is a matrix that is adjacency relevant and does not contain negative elements $a_{ij}$, where $a_{ij}>0$ is satisfied if (i,j)∈E. Otherwise, $a_{ij}=0$. $N_{i}=\left\{j|\left(i,j\right)\in E\right\}$ is defined as the set of nodes that are neighbors with node *i*, and $a_{ij}>0$ is satisfied for each $j\in N_{i}$. We denote the input matrix $D=diag\{d_{i}\}$, where $d_{i}=\sum_{j\in N}A_{ij}$. The Laplacian matrix is then defined as $L=D-A\in R^{N\times N}.$

A leader’s relationship with its followers is the subject of this article. In order to describe follower-leader interactions, we propose an enhanced directed graph model, (i.e., $\hat{G}=\left(\hat{V},\hat{E}\right)$, in which $\hat{V}=\left\{0,1,2,\ldots ,N\right\}$ and $\hat{E}\in \hat{V}\times \hat{V}$). A leader’s communication with his or her followers is determined by $b_{i}$. If $b_{i}>0$, then there is an assumption that the leader and followers are in communication. Otherwise, $b_{i}=0$. $B=diag\left\{b_{1},\ldots ,b_{n}\right\}\in R^{N\times N}$ is defined as the matrix of related connections.

### 2.2. Problem Formulation

If a nonlinear MAS has one leader as well as N followers, then the dynamics for the ith follower would be as follows:

(1) $$x_{i}\left(k+1\right)=Ax_{i}\left(k\right)+B_{i}u_{i}\left(k\right)$$

In this case, $x_{i}\in R^{N}$ represents the system state, $u_{i}\in R^{p_{i}}$ represents the control input, and $A\in R^{n\times n},B_{i}\in R^{n\times n}$ represent unknown matrices for the plants and inputs.

The leader is written as follows:

(2) $$x_{0}\left(k+1\right)=Ax_{0}\left(k\right)$$

It is assumed that $x_{0}\in R^{n}$ represents the leader state.

**Assumption** **1.** *If there is a spanning tree with a leader, then $\hat{G}$ has a network of communication interactions, and $\hat{G}$ does not contain repeated edges.*

**Definition** **1.** *As a result of our design, we are able to develop a control scheme $u_{i}\left(k\right)$ that only requires agent information. Therefore, the followers can keep track of the leader. In the event that the funder’s conditions are met, we will be able to implement a perfect control scheme [32]:*

(3) $$\lim_{k\to \infty }\left\|x_{i}\left(k\right)-x_{0}\left(k\right)\right\|=0,i=1,2,\ldots ,n$$

The MAS’s local consensus error is expressed as follows:

(4) $$e_{i}\left(k\right)=\sum_{j\in N_{i}}^{}a_{ij}\left(x_{i}\left(k\right)-x_{j}\left(k\right)\right)+b_{i}\left(x_{i}\left(k\right)-x_{0}\left(k\right)\right)$$

Then, an overview of the error vector is presented as follows:

(5) $$e\left(k\right)=\left(\left(L+B\right)⨂I_{n}\right)\left(x\left(k\right)-\hat{x_{0}}\left(k\right)\right)$$

$e\left(k\right)={\left({e_{1}}^{T}\left(k\right),{e_{2}}^{T}\left(k\right),\ldots ,{e_{n}}^{T}\left(k\right)\right)}^{T}\in R^{nN}$, $x\left(k\right)={\left({x_{1}}^{T}\left(k\right),{x_{2}}^{T}\left(k\right),\ldots ,{x_{n}}^{T}\left(k\right)\right)}^{T}\in R^{nN}$, $\hat{x_{0}}\left(k\right)=I_{n}⨂x_{0}\in R^{nN}$, as well as vector $I_{n}$ having *n* dimensions.

The tracking error is written as $\zeta_{i}\left(k\right)=x_{i}\left(k\right)-x_{0}\left(k\right)$, which has the vector form

(6) $$\zeta \left(k\right)=x\left(k\right)-\hat{x_{0}}\left(k\right)$$

In this equation, $\zeta \left(k\right)={\left({\zeta_{1}}^{T}\left(k\right),{\zeta_{2}}^{T}\left(k\right),\ldots ,{\zeta_{n}}^{T}\left(k\right)\right)}^{T}\in R^{nN},$$\hat{x_{0}}\left(k\right)={\left({x_{0}}^{T}\left(k\right),{x_{0}}^{T}\left(k\right),\ldots ,{x_{0}}^{T}\left(k\right)\right)}^{T}$.

Consequently, the localized neighbor error $e_{i}\left(k\right)$ is represented in the following manner, in agreement with Equations (1) and (4):

(7) $$\begin{array}{cc} e_{i}\left(k+1\right)= & Ae_{i}\left(k\right)+\left(d_{i}+b_{i}\right)B_{i}u_{i}\left(k\right)\\ & -\sum_{j\in N_{i}}^{}a_{ij}B_{j}u_{j}\left(k\right)\\ = & F_{i}\left(e_{i}\left(k\right),u_{i}\left(k\right)\right) \end{array}$$

Given Equations (5) and (6), it is evident that e(k) and ζ(k) are related as follows: $\lim_{k\to \infty }\left\|e\left(k\right)\right\|=0$ as $\lim_{k\to \infty }\left\|\zeta \left(k\right)\right\|=0$. Consequently, when the localized neighboring error is close to zero, the control problem is resolved.

## 3. Design of the IrQL Method

To resolve the issue of tracking control in systems with multiple agents, the authors of [32] developed the IrQL method. What is important is that in order to provide agents with a greater level of local information from other agents or environments, it is necessary to introduce IRR information, thereby improving control and learning efficiency. In addition, agents have been defined according to the Q-function, and the relevant HJB equation is acquired using the IrQL method.

As an example, consider the following IR function for the ith agent:

(8) $$\begin{array}{cc} j_{i}\left(e_{i}\left(k\right),u_{i}\left(k\right),u_{-i}\left(k\right)\right)= & e_{i}{\left(k\right)}^{T}R_{ii}e_{i}\left(k\right)+u_{i}{\left(k\right)}^{T}Q_{ii}u_{i}\left(k\right)\\ & +\sum_{j\in N_{i}}^{}u_{j}{\left(k\right)}^{T}Q_{ij}u_{j}\left(k\right) \end{array}$$

In this case, we can represent the agent’s neighbors’ input with $u_{-i}=\left\{u_{j}|j\in N_{i}\right\}$. The weight matrices $R_{ii}>0,Q_{ii}>0$, and $Q_{ij}>0$ are positive.

According to the IR function, as a function of IRR, the following is expressed:

(9) $$R_{i}\left(e_{i}\left(k\right),u_{i}\left(k\right),u_{-i}\left(k\right)\right)=\sum_{s=k}^{\infty }r^{s-k}j_{i}\left(e_{i}\left(s\right),u_{i}\left(s\right),u_{-i}\left(s\right)\right)$$

where the IRR function is defined as r∈(0,1] and *r* is its discount factor.

The following performance indices must be minimized for every agent to find a solution to the issue of controlling tracking optimally:

(10) $$J_{i}\left(e_{i}\left(0\right),u_{i}\left(0\right),u_{-i}\left(0\right)\right)=\sum_{t=0}^{\infty }\beta^{t}R_{i}\left(e_{i}\left(t\right),u_{i}\left(t\right),u_{-i}\left(t\right)\right)$$

In this case, its performance index discount factor is β∈(0,1].

**Remark** **1.** *The function of the designed IRR function incorporates accumulated prospective long-term reward data from the IR function. The performance factor is measured depending on IRR as opposed to IR, which is contrary to the majority of methods. The advantage is that we can enhance the control actions, and the learning process can be accelerated by using a great deal of data.*

**Remark** **2.** *Intrinsic motivation (IM) provides a possible method for enhancing the faculty of abstract actions or solving the difficulties associated with exploring the environment in its reinforcement learning direction. IRR acts as a driving agent that learns skills through intrinsic motivation [32].*

**Definition** **2.** *In order to resolve the MAS’s tracking control issue, we propose a distributed tracking control scheme. As the time step k approaches infinity, $e_{i}\left(k\right)⟶0$ minimizes the performance metrics (10) simultaneously.*

We can obtain a state value function as follows based on the control method of the agent as well as the neighbors $u_{i}\left(t\right)$ and $u_{-i}\left(t\right)$:

(11) $$V_{i}\left(e_{i}\left(k\right)\right)=\sum_{t=k}^{\infty }\beta^{t-k}R_{i}\left(e_{i}\left(t\right),u_{i}\left(t\right),u_{-i}\left(t\right)\right)$$

Equation (11) can also be expressed as the following formula:

(12) $$V_{i}(e_{i}\left(k\right)=R_{i}\left(e_{i}\left(k\right),u_{i}\left(k\right),u_{-i}\left(k\right)\right)+\beta V_{i}\left(e_{i}\left(k+1\right)\right)$$

Based on the theory, the ideal state value function meets the following conditions:

(13) $$\begin{array}{c} V_{i}^{*}\left(e_{i}\left(k\right)\right)=\underset{u_{i}\left(k\right)}{min}\left\{R_{i}\left(e_{i}\left(k\right),u_{i}\left(k\right),u_{-i}\left(k\right)\right)+\beta V_{i}^{*}\left(e_{i}\left(k+1\right)\right)\right\} \end{array}$$

In this case, in Bellman form, the function of IRR is expressed as

(14) $$\begin{array}{cc} R_{i}(e_{i} & \left(k\right),u_{i}\left(k\right),u_{-i}\left(k\right))\\ = & j_{i}\left(e_{i}\left(k\right),u_{i}\left(k\right),u_{-i}\left(k\right)\right)\\ & +\varrho R_{i}\left(e_{i}\left(k+1\right),u_{i}\left(k+1\right),u_{-i}\left(k+1\right)\right) \end{array}$$

On the basis of the condition of stationarity, (i.e., $\frac{\partial V_{i}^{*}\left(e_{i}\left(k\right)\right)}{\partial u_{i}\left(k\right)}$), the description of the optimal distributed control method is given below:

(15) $$\begin{array}{cc} u_{i}^{*}\left(k\right) & =arg\underset{u_{i}\left(k\right)}{min}\left\{R_{i}\left(e_{i}\left(k\right),u_{i}\left(k\right),u_{N_{i}}\left(k\right)\right)+\beta V_{i}^{*}\left(e_{i}\left(k+1\right)\right)\right\}\\ & =-\frac{1}{2}\beta \left(d_{i}+b_{i}\right)Q_{ii}^{-1}h_{i}^{T}\left(x_{i}\left(k\right)\right)▽V_{i}^{*}\left(e_{i}\left(k+1\right)\right) \end{array}$$

In this equation, $▽V_{i}^{*}\left(e_{i}\left(k+1\right)\right)=\frac{\partial V_{i}^{*}\left(e_{i}\left(k+1\right)\right)}{\partial e_{i}\left(k+1\right)}$.

**Remark** **3.** *As is well known, the state value algorithm $V_{i}\left(e_{i}\left(k\right)\right)$ is highly concerned with the space of states. In accordance with the state action function, the Q-learning method is designed with RL. The Q-function can be used by each agent to estimate the properties of all possible decisions in the current situation, and we can determine what is the best behavior of the agent at each step by using the Q-function.*

The Q-function is written as follows:

(16) $$\begin{array}{cc} Q_{i}\left(e_{i}\left(k\right),u_{i}\left(k\right),u_{-i}\left(k\right)\right)=R_{i}(e_{i}\left(k\right), & u_{i}\left(k\right),u_{-i}\left(k\right))\\ & +\beta V_{i}\left(e_{i}\left(k+1\right)\right) \end{array}$$

In accordance with the optimal scheme, the optimal Q-function is given by

(17) $$\begin{array}{cc} Q_{i}^{*} & \left(e_{i}\left(k\right),u_{i}\left(k\right),u_{-i}\left(k\right)\right)\\ = & R_{i}\left(e_{i}\left(k\right),u_{i}\left(k\right),u_{-i}\left(k\right)\right)\\ & +\beta Q_{i}^{*}\left(e_{i}\left(k+1\right),u_{i}^{*}\left(k+1\right),u_{-i}^{*}\left(k+1\right)\right) \end{array}$$

Based on Equations (16) and (17), we can express the optimal solution as follows:

(18) $$u_{i}^{*}\left(k\right)=arg\underset{u_{i}\left(k\right)}{min}\left\{Q_{i}^{*}\left(e_{i}\left(k\right),u_{i}\left(k\right),u_{-i}\left(k\right)\right)\right\}$$

In comparison with the control method of Equation (15), its optimum Q-function provides the optimal solution for the control scheme here. As a result, we intend to calculate the solution to Equation (17).

## 4. Designs of the Event-Driven Controller

According to a previous work [18], a time-triggered controller was developed. Nevertheless, a new event-triggering mechanism is designed to minimize computing costs for this case.

$Q_{i}^{*},\left(e_{i}\left(k\right),u_{i}\left(k\right),u_{-i}\left(k\right)\right)$ is defined as the sequence of trigger times. At the triggering instant, the sampled disagreement error is expressed as ${\hat{e}}_{i}^{s}$.

As a result of the threshold value and error, the triggering time varies. The control scheme can only be updated when $k=kt_{s}^{i}$ and cannot be updated under any other circumstances:

(19) $$u_{i}\left(k\right)=u_{i}\left(kt_{s}^{i}\right),k\in \left[kt_{s}^{i},kt_{s+1}^{i}\right)$$

To design a triggering condition, we propose a function that measures the gap arising from the existing error and the previously sampled error:

(20) $$\epsilon_{i}^{s}\left(k\right)={\hat{e}}_{i}^{s}-e_{i}\left(k\right),k\in \left[kt_{s}^{i},kt_{s+1}^{i}\right)$$

We have set the triggering error equal to zero at $k=kt_{s}^{i}$.

The dynamic expression of localized mistakes based on an event-triggered controlling approach can be written as

(21) $$e_{i}\left(k+1\right)=F_{i}\left(e_{i}\left(k\right),u_{i}\left(kt_{s}^{i}\right)\right)$$

Thus, the equation for event-triggered events is obtained:

(22) $$\begin{array}{cc} V_{i}^{*} & (e_{i}\left(k\right))=\\ & \underset{u_{i}\left(kt_{s}^{i}\right)}{min}\left\{R_{i}\left(e_{i}\left(k\right),u_{i}\left(kt_{s}^{i}\right),u_{-i}\left(kt_{s}^{i}\right)\right)+\beta V_{i}^{*}\left(F_{i}\left(e_{i}\left(k\right),u_{i}\left(kt_{s}^{i}\right)\right)\right)\right\} \end{array}$$

(23) $$\begin{array}{c} Q_{i}^{*}(e_{i}\left(k\right))\\ \;=R_{i}\left(e_{i}\left(k\right),u_{i}\left(kt_{s}^{i}\right),u_{-i}\left(kt_{s}^{i}\right)\right)\\ \;+\beta Q_{i}^{*}\left(F_{i}\left(e_{i}\left(k\right),u_{i}\left(kt_{s}^{i}\right)\right)\right) \end{array}$$

It is possible to express the optimal tracking control using an event-triggered approach in the following way:

(24) $$u_{i}^{*}\left(k\right)=arg\underset{u_{i}\left(kt_{s}^{i}\right)}{min}\left\{Q_{i}^{*}\left(e_{i}\left(k\right)\right)\right\}$$

**Assumption** **2.** *There is a constant L that explains the inequality below:*

(25) $$∥F_{i}\left(e_{i}\left(k\right),u_{i}\left(kt_{s}^{i}\right)\right)∥\leq L∥e_{i}\left(k\right)∥+L∥\epsilon_{i}^{s}\left(k\right)∥$$

**Assumption** **3.** *There is a triggering condition which is as follows:*

(26) $${∥\epsilon_{i}^{s}\left(k\right)∥}^{2}\leq \left(1-2L^{2}\right)/\left(2L^{2}\right){∥e_{i}\left(k\right)∥}^{2}=\pi_{i}T$$

*where $\pi_{i}T$ represents the triggering threshold and $L\in (0,\sqrt{2}/2)$ [24]. Once the multi-agent system dynamics have stabilized, followers are able to track their leaders.*

## 5. Neural Network Implementation for the Event-Triggered Approach Using the IrQL Method

This section discusses the tree-NN structure, also known as RCA-NNs. Three virtual networks are included in the tree-NN structure.

### 5.1. Reinforce Neutral Network (RNN) Learning Model

The reinforced NN is employed to approximate the IRR signal as follows:

(27) $$\hat{R}\left(Z_{ri}\left(k\right)\right)=\varphi_{ri}\left(\omega_{r2i}^{T}\left(k\right)\cdot \varphi_{ri}\left(\omega_{r1i}^{T}\left(k\right)\cdot Z_{ri}\left(k\right)\right)\right)$$

where $Z_{ri}\left(k\right)$ represents the input vector, which has $e_{i}\left(k\right),$$u_{i}\left(k\right)$, while $u_{-i}\left(k\right)$. $\omega_{r1i}$ represents the matrix of weights for input-to-hidden layering. Meanwhile, $\omega_{r2i}$ represents the matrix of weights for hidden-to-output layering, and $\varphi_{ri}\left(\cdot \right)$ represents an activation function [24].

Due to the reinforced NN, the associated error function is as follows:

(28) $$\begin{array}{cc} e_{ri}\left(k\right)=j_{i}(e_{i}\left(k-1\right), & u_{i}\left(k-1\right),u_{-i}\left(k-1\right))\\ & +\varrho {\hat{R}}_{i}\left(Z_{ri}\left(k\right)\right)-{\hat{R}}_{i}\left(Z_{ri}\left(k-1\right)\right) \end{array}$$

The loss function is written as

(29) $$E_{ri}\left(k\right)=\frac{1}{2}e_{ri}^{2}\left(k\right)$$

For convenience’s sake, only the matrices $\omega_{r2i}$ are updated, and the matrices $\omega_{r1i}$ remain unchanged during the training process.

The RNN’s update law is expressed as

(30) $$\omega_{r2i}\left(k+1\right)=\omega_{r2i}\left(k\right)-\alpha_{ri}\cdot \left(\frac{\partial E_{ri}\left(k\right)}{\partial \omega_{r2i}\left(k\right)}\right)$$

In this equation, $\alpha_{ri}$ represents the rate at which the RNN learns.

The gradient descent rule (GDR) is used to obtain an updated law for the reinforced NN’s weight, which yields the following results:

(31) $$\begin{array}{cc} \; & \omega_{ri}\left(k+1\right)\\ & \;=\omega_{ri}\left(k\right)-\alpha_{ri}\cdot \left(\frac{\partial E_{ri}\left(k\right)}{\partial e_{ri}\left(k\right)}\cdot \frac{\partial e_{ri}\left(k\right)}{\partial \hat{R}\left(Z_{ri}\left(k\right)\right)}\cdot \frac{\partial \hat{R}\left(Z_{ri}\left(k\right)\right)}{\partial \omega_{r2i}\left(k\right)}\right)\\ & \;=\omega_{r2i}\left(k\right)-\alpha_{ri}\varrho e_{ri}\left(k\right)\left[1-\varphi_{ri}^{2}\left(\omega_{r2i}^{T}\left(k\right)\cdot \Delta_{ri}\left(k\right)\right)\right]\Delta_{ri}\left(k\right) \end{array}$$

In this equation, $\Delta_{ri}\left(k\right)=\varphi_{ri}\left(\omega_{r1i}^{T}\left(k\right)\cdot Z_{ri}\left(k\right)\right)$.

### 5.2. Critic Neutral Network (CNN) Learning Model

In the following section, when designing the critic NN, an attempt is made to achieve a close approximation of the Q-function:

(32) $$\hat{Q_{i}}\left(Z_{ci}\left(k\right)\right)=\omega_{c2i}^{T}\left(k\right)\cdot \varphi_{ci}\left(\omega_{c1i}^{T}\left(k\right)\cdot Z_{ci}\left(k\right)\right)$$

In this equation, $Z_{ci}\left(k\right)$ represents the relative vector of inputs that has ${\hat{R}}_{i}\left(k\right)$, $e_{i}\left(k\right)$, and $u_{i}\left(k\right)$ as well as $u_{-i}\left(k\right)$, while $\omega_{c1i}^{T}\left(k\right)$ and $\omega_{c2i}^{T}\left(k\right)$ represent the input layer weight matrices and output layer weight matrices.

It is possible to express the function of the error for the CNN to be

(33) $$e_{ci}\left(k\right)={\hat{R}}_{i}\left(Z_{ri}\left(k-1\right)\right)+\beta {\hat{Q}}_{i}\left(Z_{ci}\left(k\right)\right)-{\hat{Q}}_{i}\left(Z_{ci}\left(k-1\right)\right)$$

Its function of loss is written to be

(34) $$E_{ci}\left(k\right)=\frac{1}{2}e_{ci}^{2}\left(k\right)$$

In accordance with the operation of RNNs, only $\omega_{c2i}$ is updated, and $\omega_{c1i}$ remains unchanged.

With the help of the gradient descent rule (GDR), it can be used to express the weight update law:

(35) $$\omega_{c2i}\left(k+1\right)=\omega_{c2i}\left(k\right)-\alpha_{ci}\left(\frac{\partial E_{ci}\left(k\right)}{\partial \omega_{c2i}\left(k\right)}\right)$$

where $\alpha_{ci}$ represents the critic NN’s learning rate. Furthermore, we can obtain its weight update schemes for the critic NN:

(36) $$\begin{array}{cc} \; & \omega_{c2i}\left(k+1\right)\\ & \;=\omega_{c2i}\left(k\right)-\alpha_{ci}\left(\frac{\partial E_{ci}\left(k\right)}{\partial e_{ci}\left(k\right)}\cdot \frac{\partial e_{ci}\left(k\right)}{\partial {\hat{Q}}_{i}\left(Z_{ci}\left(k\right)\right)}\cdot \frac{\partial {\hat{Q}}_{i}\left(Z_{ci}\left(k\right)\right)}{\partial \omega_{c2i}\left(k\right)}\right)\\ & \;=\omega_{c2i}\left(k\right)-\alpha_{ci}\beta \left[{\hat{R}}_{i}\left(Z_{ci}\left(k-1\right)\right)+\beta \omega_{c2i}^{T}\left(k\right)\cdot \Delta_{ci}\left(k\right)\right.\\ \; & \left.\;-\omega_{c2i}^{T}\left(k-1\right)\cdot \Delta_{ci}\left(k-1\right)\right]\cdot \Delta_{ci}\left(k\right) \end{array}$$

In this equation, $\Delta_{ci}\left(k\right)=\varphi_{ci}\left(\omega_{ci1}^{T}\left(k\right)Z_{ci}\left(k\right)\right)$.

### 5.3. Actor Neutral Network (ANN) Learning Model

Based on the actor NN, an approximate optimal scheme is defined as follows:

(37) $${\hat{u}}_{i}\left(k\right)=\omega_{a2i}^{T}\cdot \varphi_{ai}\left(\omega_{a1i}^{T}\cdot Z_{ai}\left(k\right)\right)$$

where the input data of the ANN is represented by $Z_{ai}\left(k\right)=e_{i}\left(k\right)$, $\omega_{a1i}$ represents the weight matrices of the input layer, and $\omega_{a2i}$ represents the weight matrices of the output layer.

Based on the prediction error of the actor NN, the following result is obtained:

(38) $$e_{ai}\left(k\right)={\hat{Q}}_{i}\left(Z_{ci}\left(k\right)\right)-U_{c}$$

It is possible to express the function of loss of the ANN to be

(39) $$E_{ai}\left(k\right)=\frac{1}{2}e_{ai}\left(k\right)$$

As with RNNs and CNNs, $\omega_{a1i}$ must remain unchanged throughout the learning process. The actor NN update laws are defined as follows:

(40) $$\omega_{a2i}\left(k+1\right)=\omega_{a2i}\left(k\right)-\alpha_{ai}\left(\frac{\partial E_{ai}\left(k\right)}{\partial \omega_{a2i}\left(k\right)}\right)$$

where $\alpha_{ai}$ represents the ANN learning rate. We can design a weight-tuning scheme for an ANN as follows:

(41) $$\begin{array}{cc} \; & \omega_{a2i}\left(k+1\right)\\ \; & \;=\omega_{a2i}\left(k\right)-\alpha_{ai}\cdot \left(\frac{\partial E_{ai}\left(k\right)}{\partial e_{ai}\left(k\right)}\cdot \frac{\partial e_{ai}\left(k\right)}{\partial {\hat{Q}}_{i}\left(Z_{ci}\left(k\right)\right)}\right.\\ \; & \left.\;\times \frac{\partial {\hat{Q}}_{i}\left(Z_{ci}\left(k\right)\right)}{\partial {\hat{u}}_{i}\left(k\right)}\cdot \frac{\partial {\hat{u}}_{i}\left(k\right)}{\partial \omega_{a2i}\left(k\right)}\right)\\ \; & \;=\omega_{a2i}\left(k\right)-\alpha_{ai}\Delta_{ai}\left(k\right)\omega_{a2i}^{T}\left(k\right)\\ \; & \;\times {▽}_{ci}^{{}^{\prime }}\left(k\right)\omega_{c1i}^{T}\left(k\right){▽}_{{\hat{u}}_{i}}\left(Z_{ci}\left(k\right)\right)\left[\omega_{a2i}^{T}\Delta_{ci}\left(k\right)\right] \end{array}$$

where $\Delta_{ai}\left(k\right)=\varphi_{ai}\left(\omega_{a1i}^{T}\left(k\right)Z_{ai}\left(k\right)\right)$, ${▽}_{ci}^{{}^{\prime }}\left(k\right)=\frac{\partial \varphi_{ci}\left(\omega_{c1i}\left(k\right)Z_{ci}\left(k\right)\right)}{\partial \varphi_{ci}\left(\omega_{c1i}^{T}\left(k\right)Z_{ci}\left(k\right)\right)}$, ${▽}_{{\hat{u}}_{i}}\left(Z_{ci}\left(k\right)\right)=\frac{\partial Z_{ci}\left(k\right)}{\partial {\hat{u}}_{i}\left(k\right)}$.

Furthermore, we can obtain

(42) $$\omega_{a2i}\left(k+1\right)=\left\{\begin{array}{c} \omega_{a2i}\left(k\right)-\alpha_{ai}\Delta_{ai}\left(k\right)\omega_{a2i}^{T}\left(k\right)\\ \times {▽}_{ci}^{{}^{\prime }}\left(k\right)\omega_{c1i}^{T}\left(k\right){▽}_{{\hat{u}}_{i}}\left(Z_{ci}\left(k\right)\right)\left[\omega_{a2i}^{T}\Delta_{ci}\left(k\right)\right],k=kt_{s}^{i}\\ \omega_{a2i}\left(k\right),k\in \left[kt_{s}^{i},kt_{s+1}^{i}\right). \end{array}\right.$$

It is described in detail in Algorithm 1 how the controller is designed using RCA-NNs and event triggering. When the trigger conditions are met, the actor NN is updated.

For analysis of stability based on the Lyapunov method, we present an analysis of stability and convergence in the following section.

**Assumption** **4.** *The following conditions are assumed to be true: $∥\omega_{r2i}\left(k\right)∥\leq \omega_{rim},∥\omega_{c2i}\left(k\right)∥$$\leq \omega_{cim},∥\omega_{a2i}\left(k\right)∥\leq \omega_{aim}$. There are bounded activation functions, i.e., $∥\Delta_{ri}\left(k\right)∥\leq \Delta_{rim},∥\Delta_{ci}\left(k\right)∥\leq \Delta_{cim},∥\Delta_{ai}\left(k\right)∥\leq \Delta_{aim}$. What’s more, the functions of activation $\varphi_{ai}\left(k\right)$ is the function of Lipschitz that satisfies $∥\varphi_{ai}\left(e_{i}\left(kt_{s}^{i}\right)\right)-\varphi_{ai}\left(k\right)∥\leq \theta_{ai}∥e_{i}\left(kt_{s}^{i}\right)-e_{i}\left(k\right)∥=\theta_{ai}∥\epsilon_{i}^{s}\left(k\right)∥\leq \theta_{ai}\pi_{i}T$, where $\theta_{ai}$, $\pi_{i}T$ are positive constants. Approximation errors of NNs’ output can be defined to be: $\delta_{ci}\left(k\right)=\omega_{c2i}\left(k\right)\Delta_{ci}\left(k\right),\delta_{ai}\left(k\right)=\omega_{a2i}\left(k\right)\Delta_{ai}\left(k\right),\vartheta_{ri}\left(k\right)=\omega_{r2i}\left(k\right)\Delta_{ri}\left(k\right)$.*

**Theorem** **1.** *Assume that Assumptions 1 and 2 are true. CNN and ANN weights are renewed by (36) and (42). Upon satisfying the triggering term(26), the local inconsistency error is $e_{i}\left(k\right)$, critic evaluated error and actor evaluated error error are consistent and ultimately bounded. Furthermore the control method $u_{i}$ converges to the optimal value $u_{i}^{*}$.*

Evidence: Set ${\tilde{\omega }}_{r2i}\left(k\right)=\omega_{r2i}\left(k\right)-\omega_{r2i}^{*}$ as the weighting assessment error between the optimal weights for RNNs $\omega_{r2i}^{*}$. Its assessment $\omega_{r2i}\left(k\right)$, ${\tilde{\omega }}_{c2i}\left(k\right)=\omega_{c2i}\left(k\right)-\omega_{c2i}^{*}$ is the error resulting from weighting evaluation involving the ideal CNN weights $\omega_{c2i}^{*}$; its assessed $\omega_{c2i}\left(k\right)$, as well as ${\tilde{\omega }}_{a2i}\left(k\right)=\omega_{a2i}\left(k\right)-\omega_{a2i}^{*}$ is the weighting evaluated error involving the ideal ANN weightings $\omega_{a2i}^{*}$ and its estimation $\omega_{a2i}\left(k\right)$.
**Algorithm 1** RCA neural networks based on the IrQL method with event triggering.Set initial value:1: Set initial values for $\omega_{r2i}\left(0\right),\omega_{a2i}\left(0\right),\omega_{c2i}\left(0\right)$ between (0,1);2: Set a low level of degree of precision for the calculation E.3: Initialize the score of $x_{i}\left(0\right),x_{0}\left(0\right)$ within (0,1)The iterative process: Make kisequalto0. Error calculation at the localized level $e_{i}\left(k\right)$;4: Keep on;5: Based on actor NN, estimate ${\hat{u}}_{i}\left(k\right)$ by (37)6: Update the reinforce NN;7: Via the inputting $\left[e_{i}\left(k\right),u_{i}\left(k\right),u_{-i}\left(k\right)\right]$ into the reinforce NN, and we can obtain theestimated the function of IRR $R_{i}\left(Z_{ri}\left(k\right)\right)$ via (27)8: Obtain $e_{ri}\left(k\right)$ by (28);9: Renew the matrices $\omega_{r2i}\left(k\right)$ by (31);10: Renew the critic NN:11: Via the inputting $\left[{\hat{R}}_{i}\left(Z_{ri}\left(k\right)\right),e_{i}\left(k\right),u_{i}\left(k\right),andu_{-i}\left(k\right)\right]$ into critic NN,and we can obtain its estimated Q-function via (32);12: Obtain $e_{ci}\left(k\right)$ by (33);13: Renew the matrices $\omega_{c2i}\left(k\right)$ by (36);14: Renew the actor NN:15: Input $\left[e_{i}\left(k\right)\right]$ to the actor NN, and we can obtain the estimated Q-function${\hat{u}}_{i}\left(k\right)$ via (37)16: Calculation $e_{ai}\left(k\right)$ via (38)17: In the event that the triggering conditions are met, renew the matrices$\omega_{a2i}\left(k\right)$ of the actor NN using (41)18: Otherwise, do not update the weight matrices $\omega_{a2i}\left(k\right)$19: Until $∥\omega_{c2i}\left(k+1\right)-\omega_{c2i}\left(k\right)∥\leq E$; otherwise, set k=k+1, then go toprocedure (5)20: Keep on $\omega_{r2i}\left(k\right),\omega_{c2i}\left(k\right),\omega_{a2i}\left(k\right)$ as the optimal weights.

(1) We can obtain the following function at the time of triggering as follows:

(43) $$L\left(k\right)=L_{1}\left(k\right)+L_{2}\left(k\right)+L_{3}\left(k\right)+L_{4}\left(k\right)+L_{5}\left(k\right)$$

In this equation,

(44) $$\begin{array}{cc} \; & L_{1}\left(k\right)=\frac{1}{\alpha_{ri}}tr\left(\omega_{r2i}^{T}\left(k\right)\omega_{r2i}\left(k\right)\right),\\ \; & L_{2}\left(k\right)=\frac{1}{\alpha_{ci}}tr\left(\omega_{c2i}^{T}\left(k\right)\omega_{c2i}\left(k\right)\right),\\ \; & L_{3}\left(k\right)=\frac{1}{\alpha_{ai}}tr\left(\omega_{a2i}^{T}\left(k\right)\omega_{a2i}\left(k\right)\right),\\ \; & L_{4}\left(k\right)=\varrho^{k}{\hat{R}}_{i}\left(k\right),\\ \; & L_{5}\left(k\right)=\beta^{k}{\hat{Q}}_{i}\left(k\right). \end{array}$$

$\Delta L_{1}\left(k\right)$ is written to be

(45) $$\Delta L_{1}\left(k\right)=\frac{1}{\alpha_{ri}}tr\left(\omega_{r2i}^{T}\left(k+1\right)\omega_{r2i}\left(k+1\right)-\omega_{r2i}^{T}\left(k\right)\omega_{r2i}\left(k\right)\right).$$

In this equation, we have

(46) $$\begin{array}{cc} {\tilde{\omega }}_{r2i}\left(k+1\right) & =\omega_{r2i}\left(k+1\right)-\omega_{r2i}^{*}\\ \; & =\omega_{r2i}\left(k\right)-\alpha_{ri}\varrho \left[j\left(k-1\right)+\varrho \hat{R}\left(k\right)-\hat{R}\left(k-1\right)\right]\\ & \times \delta_{ri}\left(k\right)\Delta_{ri}\left(k\right) \end{array}$$

Furthermore, we have

(47) $$\begin{array}{cc} \Delta L_{1}\left(k\right)= & -2\varrho^{2}\delta_{ri}\left(k\right)\left[\varrho^{-1}j\left(k\right)+\hat{R}\left(k\right)-\hat{R}\left(k-1\right)\right]\\ \; & +\alpha_{ri}\varrho^{4}{\left[\varrho^{-1}j\left(k\right)+\hat{R}\left(k\right)-\hat{R}\left(k-1\right)\right]}^{2}\vartheta_{ri}^{2}\left(k\right)\\ = & {\left\{\delta_{ri}\left(k\right)-\varrho^{2}\left[\varrho^{-1}j\left(k\right)+\hat{R}\left(k\right)-\hat{R}\left(k-1\right)\right]\right\}}^{2}\\ \; & -\left(1-\alpha_{ri}\left(k\right)\Delta_{ri}^{2}\left(k\right)\right)\varrho^{4}{\left[\varrho^{-1}j\left(k\right)+\hat{R}\left(k\right)-\hat{R}\left(k-1\right)\right]}^{2}\\ \; & \times \vartheta_{ri}^{2}\left(k\right)-\delta_{ri}^{2}\left(k\right) \end{array}$$

$\Delta L_{2}\left(k\right)$ can be written as

(48) $$\Delta L_{2}\left(k\right)=\frac{1}{\alpha_{ci}}tr\left(\omega_{c2i}^{T}\left(k+1\right)\omega_{c2i}\left(k+1\right)-\omega_{c2i}^{T}\left(k\right)\omega_{c2i}\left(k\right)\right).$$

Within this equation, we have

(49) $$\begin{array}{cc} {\tilde{\omega }}_{c2i}\left(k+1\right) & =\omega_{c2i}\left(k+1\right)-\omega_{c2i}^{*}\\ \; & =\omega_{c2i}\left(k\right)-\alpha_{ci}\beta \Delta_{ci}\left(k\right)[{\hat{R}}_{i}\left(k-1\right)\\ & +\beta \left(\omega_{c2i}\left(k\right)+\omega_{c2i}^{*}\right)\Delta_{ci}\left(k\right)-\omega_{c2i}^{T}\left(k-1\right)\Delta_{ci}\left(k-1\right)] \end{array}$$

Furthermore, we have

(50) $$\Delta L_{2}\left(k\right)=\frac{1}{\alpha_{ci}}\left\{D_{1}+D_{2}+D_{3}-\omega_{c2i}^{T}\left(k\right)\omega_{c2i}\left(k\right)\right\}$$

where

$$\begin{array}{cc} D_{1} & =\omega_{c2i}^{T}\left(k\right){\left(I-\alpha_{ci}\beta^{2}\Delta_{ci}\left(k\right)\Delta_{ci}^{T}\left(k\right)\right)}^{2}\omega_{c2i}(k\\ \; & ={∥\omega_{c2i}\left(k\right)∥}^{2}-2\alpha_{ci}\beta^{2}{∥\delta_{ci}\left(k\right)∥}^{2}\\ \; & +\alpha_{ci}^{2}\beta^{4}{∥\Delta_{ci}\left(k\right)∥}^{2}{∥\delta_{ci}\left(k\right)∥}^{2}\\ D_{2} & =-2\alpha_{ci}\beta^{2}\delta_{ci}\left(k\right)[\beta^{-1}{\hat{R}}_{i}\left(k-1\right)+{\left(\omega_{c2i}^{*}\right)}^{T}\Delta_{ci}\left(k\right)\\ \; & -\beta^{-1}\omega_{c2i}\left(k-1\right)\Delta_{ci}\left(k-1\right)]{∥\Delta_{ci}\left(k\right)∥}^{2}\\ D_{3} & =\alpha_{ci}^{2}\beta^{4}[\beta^{-1}{\hat{R}}_{i}\left(k-1\right)+{\left(\omega_{c2i}^{*}\right)}^{T}\Delta_{ci}\left(k\right)\\ \; & -\beta^{-1}\omega_{c2i}^{T}\left(k-1\right)\Delta_{ci}{\left(k-1\right)]}^{T}\\ \; & \times [\beta^{-1}{\hat{R}}_{i}\left(k-1\right)+{\left(\omega_{c2i}^{*}\right)}^{T}\Delta_{ci}\left(k\right)-\beta^{-1}\omega_{c2i}^{T}\left(k-1\right)\Delta_{ci}\left(k-1\right)] \end{array}$$

The following result is obtained by computation:

(51) $$\begin{array}{cc} \Delta L_{2}\left(k\right) & =-\beta^{2}{∥\delta_{ci}\left(k\right)∥}^{2}-\beta^{2}\left(1-\alpha_{ci}\beta^{2}{∥\Delta_{ci}\left(k\right)∥}^{2}\right)\\ \; & \times \left|\right|\delta_{ci}\left(k\right)+\beta^{-1}{\hat{R}}_{i}\left(k-1\right)+{\left(\omega_{c2i}^{*}\right)}^{T}\Delta_{ci}\left(k\right)\\ \; & -\beta^{-1}\omega_{c2i}^{T}\left(k-1\right)\Delta_{ci}{\left(k-1\right))||}^{2}\\ \; & +\left|\right|{\hat{R}}_{i}\left(k-1\right)+\beta {\left(\omega_{c2i}^{*}\right)}^{T}\Delta_{ci}\left(k\right)\\ \; & -\omega_{c2i}^{T}\left(k-1\right)\Delta_{ci}\left(k-1\right){\left|\right|}^{2} \end{array}$$

In the case of the difference of the first order of $L_{3}\left(k\right)$, we can obtain

(52) $$\Delta L_{3}\left(k\right)=\frac{1}{\alpha_{ai}}\left(\omega_{a2i}^{T}\left(k+1\right)\omega_{a2i}\left(k+1\right)-\omega_{a2i}^{T}\left(k\right)\omega_{a2i}\left(k\right)\right)$$

where,

(53) $$\begin{array}{cc} {\tilde{\omega }}_{a2i}\left(k+1\right) & =\omega_{a2i}\left(k+1\right)-\omega_{a2i}^{*}\\ \; & =\omega_{a2i}\left(k\right)-\alpha_{ai}\Delta_{ai}\left(k\right)\omega_{c2i}^{T}\left(k\right)C\left(k\right)\\ \; & \times [\omega_{c2i}^{T}\left(k\right)\Delta_{ci}\left(k\right)] \end{array}$$

Therefore, we have

(54) $$\begin{array}{c} \Delta L_{3}\left(k\right)=\frac{1}{\alpha_{ai}}\left(E1-\omega_{a2i}^{T}\left(k\right)\omega_{a2i}\left(k\right)\right) \end{array}$$

where

(55) $$\begin{array}{cc} E1= & \left|\right|\omega_{a2i}\left(k\right){\left|\right|}^{2}-2\alpha_{ai}\omega_{c2i}^{T}\left(k\right)C\left(k\right)\delta_{ai}\left(k\right)\left[\omega_{c2i}^{T}\left(k\right)\Delta_{ci}\left(k\right)\right]\\ \; & +\alpha_{ai}\left|\right|\omega_{c2i}^{T}\left(k\right)\Delta_{ci}{\left(k\right)||}^{2}\left|\right|\Delta_{ai}{\left(k\right)||}^{2}\left|\right|\omega_{c2i}^{T}\left(k\right)C\left(k\right){\left|\right|}^{2} \end{array}$$

In the case of $\Delta L_{3}\left(k\right)$, the simplified formula is given below:

(56) $$\begin{array}{cc} \Delta L_{3}\left(k\right)= & -(1-\alpha_{ai}\left|\right|\Delta_{ai}{\left(k\right)||}^{2})||\omega_{c2i}^{T}\left(k\right)\Delta_{ci}\left(k\right){\left|\right|}^{2}\\ \; & \times \left|\right|\omega_{c2i}^{T}{\left(k\right)C\left(k\right)||}^{2}-\left|\right|\delta_{ai}\left(k\right){\left|\right|}^{2}\\ \; & +\left|\right|\omega_{c2i}^{T}\left(k\right)C\left(k\right)\Delta_{ai}\left(k\right)\omega_{c2i}^{T}\left(k\right)-\delta_{ai}\left(k\right){\left|\right|}^{2} \end{array}$$

By adding Equations (47), (51), and (56), we can obtain L(k) as follows:

(57) $$\begin{array}{cc} \Delta L(k) & =\Delta L_{1}\left(k\right)+\Delta L_{2}\left(k\right)+\Delta L_{3}\left(k\right)+\Delta L_{4}\left(k\right)+\Delta L_{5}\left(k\right)\\ \; & =-\beta^{2}\left|\right|\delta_{ci}{\left(k\right)||}^{2}-\beta^{2}(1-\alpha_{ci}\beta^{2}\left|\right|\Delta_{ci}{\left(k\right)||}^{2})\\ \; & \times \left|\right|\delta_{ci}\left(k\right)+\beta^{-1}{\hat{R}}_{i}\left(k-1\right)+{\left(\omega_{c2i}^{*}\right)}^{T}\Delta_{ci}\left(k\right)\\ \; & -\beta^{-1}\omega_{c2i}^{T}\left(k-1\right)\Delta_{ci}\left(k-1\right){\left|\right|}^{2}\\ \; & -(1-\alpha_{ai}||\Delta_{ai}\left(k\right)|{|}^{2})\\ \; & \times \left|\right|\omega_{c2i}^{T}\left(k\right)\Delta_{ci}{\left(k\right)||}^{2}\left|\right|\omega_{c2i}^{T}{\left(k\right)C\left(k\right)||}^{2}+\left|\right|{\hat{R}}_{i}\left(k-1\right)\\ \; & +\beta {\left(\omega_{c2i}^{*}\right)}^{T}\Delta_{ci}\left(k\right)-\omega_{c2i}^{T}\left(k-1\right)\Delta_{ci}\left(k-1\right){\left|\right|}^{2}\\ \; & +\left|\right|\omega_{c2i}^{T}\left(k\right)C\left(k\right)\Delta_{ci}^{T}\left(k\right)\omega_{c2i}^{T}\left(k\right)-\delta_{ai}\left(k\right){\left|\right|}^{2}\\ \; & -\left(1-\alpha_{ri}\Delta_{ri}^{2}\left(k\right)\right)\varrho^{4}\\ \; & \times \left|\right|\varrho^{-1}j\left(k\right)+{\hat{R}}_{i}\left(k\right)-\varrho^{-1}{\hat{R}}_{i}\left(k-1\right){\left|\right|}^{2}\\ \; & \vartheta^{2}\left(k\right)+\left|\right|\delta_{ri}\left(k\right)-\varrho^{2}[\varrho^{-1}j\left(k\right)+{\hat{R}}_{i}\left(k\right)\\ \; & -\varrho^{-1}{\hat{R}}_{i}{\left(k-1\right)]||}^{2}\\ \; & -\left|\right|\delta_{ai}{\left(k\right)||}^{2}-\left|\right|\delta_{ri}\left(k\right){\left|\right|}^{2}+\beta^{k+1}Q_{i}\left(k+1\right)-\beta^{k}Q_{i}\left(k\right)\\ \; & +\varrho^{k+1}R_{i}\left(k+1\right)-\varrho^{k}R_{i}\left(k\right) \end{array}$$

Therefore, we can obtain

(58) $$\begin{array}{cc} \Delta L(k) & =-\beta^{2}\left|\right|\delta_{ci}{\left(k\right)||}^{2}-\beta^{2}(1-\alpha_{ci}\beta^{2}\left|\right|\Delta_{ci}{\left(k\right)||}^{2})\times ||\delta_{ci}\left(k\right)\\ \; & +\beta^{-1}V_{1}{\left(k\right)||}^{2}-(1-\alpha_{ai}\left|\right|\Delta_{ai}{\left(k\right)||}^{2})||X_{1}\left(k\right){\left|\right|}^{2}\\ \; & \times \left|\right|W_{1}{\left(k\right)||}^{2}+\left|\right|V_{1}{\left(k\right)||}^{2}+\left|\right|W^{1}\left(k\right)X_{1}^{T}\left(k\right)-\delta_{ai}\left(k\right){\left|\right|}^{2}\\ \; & -(1-\alpha_{ai}\left(k\right)||\Delta_{ri}{\left(k\right)||}^{2})\varrho^{4}\left|\right|\varrho^{-1}Y_{1}\left(k\right){\left|\right|}^{2}\upsilon_{ri}^{2}\left(k\right)\\ \; & -\left|\right|\delta_{ai}{\left(k\right)||}^{2}-\left|\right|\delta_{ri}\left(k\right){\left|\right|}^{2}+\beta^{k+1}Q_{i}\left(k+1\right)-\beta^{k}Q_{i}\left(k\right)\\ \; & +\varrho^{k+1}R_{i}\left(k+1\right)-\varrho^{k}R_{i}\left(k\right) \end{array}$$

where $V_{1}\left(k\right)={\hat{R}}_{i}\left(k-1\right)+\beta {\left(\omega_{c2i}^{*}\right)}^{T}\Delta_{ci}\left(k\right)-\omega_{c2i}^{T}\left(k-1\right)\Delta_{ci}\left(k-1\right),W_{1}\left(k\right)=\omega_{c2i}^{T}\left(k\right)C\left(k\right),$$X_{1}\left(k\right)=\omega_{c2i}^{T}\left(k\right)\Delta_{ci}\left(k\right),Y_{1}\left(k\right)=j\left(k\right)+\varrho {\hat{R}}_{i}\left(k\right)-{\hat{R}}_{i}\left(k-1\right)$, and we can obtain $\left|\right|V_{1}\left(k\right)||\leq V_{1m},||W_{1}\left(k\right)||\leq W_{1m},||X_{1}\left(k\right)||\leq X_{1m},||Y_{1}\left(k\right)\left|\right|\leq Y_{1m}$. Next, we can obtain

(59) $$\begin{array}{cc} \Delta L(k)\leq & -\beta^{2}\left|\right|\delta_{ci}{\left(k\right)||}^{2}-\beta^{2}(1-\alpha_{ci}\beta^{2}\left|\right|\Delta_{ci}{\left(k\right)||}^{2})\\ \; & \times \left|\right|\delta_{ci}\left(k\right)+\beta^{-1}V_{1}\left(k\right){\left|\right|}^{2}\\ \; & -(1-\alpha_{ai}\left|\right|\Delta_{ai}{\left(k\right)||}^{2})||X_{1}{\left(k\right)||}^{2}\left|\right|W_{1}\left(k\right){\left|\right|}^{2}\\ \; & +2||W_{1}\left(k\right)X_{1}^{T}{\left(k\right)||}^{2}+\left|\right|\delta_{ai}\left(k\right){\left|\right|}^{2}\\ \; & -(1-\alpha_{ri}\left|\right|\Delta_{ri}{\left(k\right)||}^{2})\varrho^{2}\left|\right|Y_{1}\left(k\right){\left|\right|}^{2}\vartheta_{ri}^{2}\left(k\right)\\ \; & +2||\delta_{ri}{\left(k\right)||}^{2}+2||Y_{1}\left(k\right){\left|\right|}^{2}\\ \; & -\beta^{k}Q_{i}\left(k\right)-\varrho^{k}R_{i}\left(k\right) \end{array}$$

Moreover, we can obtain

(60) $$\begin{array}{cc} \Delta L(k)\leq & -\beta^{2}\left|\right|\delta_{ci}{\left(k\right)||}^{2}-\beta^{2}(1-\alpha_{ci}\beta^{2}\left|\right|\Delta_{ci}{\left(k\right)||}^{2})\\ \; & \times \left|\right|\delta_{ci}\left(k\right)+\beta^{-1}V_{1}\left(k\right){\left|\right|}^{2}\\ \; & -(1-\alpha_{ai}\left|\right|\Delta_{ai}{\left(k\right)||}^{2})||X_{1}{\left(k\right)||}^{2}\left|\right|W_{1}\left(k\right){\left|\right|}^{2}\\ \; & +V_{1m}^{2}+2W_{1m}^{2}X_{1m}^{2}+2||{\left(\omega_{a2i}^{*}\right)}^{T}\Delta_{ai}\left(k\right){\left|\right|}^{2}\\ \; & +2||\omega_{a2i}^{T}\Delta_{ai}\left(k\right){\left|\right|}^{2}\\ \; & -(1-\alpha_{ri}\left|\right|\Delta_{ri}{\left(k\right)||}^{2})\varrho^{2}\left|\right|Y_{1}\left(k\right){\left|\right|}^{2}\vartheta_{ri}^{2}\left(k\right)\\ \; & +2||\delta_{ri}{\left(k\right)||}^{2}+2||Y_{1}\left(k\right){\left|\right|}^{2}\\ \; & -\beta^{k}Q_{i}\left(k\right)-\varrho^{k}R_{i}\left(k\right)\\ \; & \leq -\beta^{2}\left|\right|\delta_{ci}{\left(k\right)||}^{2}-\beta^{2}(1-\alpha_{ci}\beta^{2}\left|\right|\Delta_{ci}{\left(k\right)||}^{2})\\ \; & \times \left|\right|\delta_{ci}\left(k\right)+\beta^{-1}V_{1}\left(k\right){\left|\right|}^{2}\\ \; & -(1-\alpha_{ai}\left|\right|\Delta_{ai}{\left(k\right)||}^{2})||X_{1}{\left(k\right)||}^{2}\left|\right|W_{1}\left(k\right){\left|\right|}^{2}\\ \; & +V_{1m}^{2}+2W_{1m}^{2}X_{1m}^{2}+4\omega_{aim}^{2}\Delta_{aim}^{2}\\ \; & -(1-\alpha_{ri}\left|\right|\Delta_{ri}{\left(k\right)||}^{2})\varrho^{2}\left|\right|Y_{1}\left(k\right){\left|\right|}^{2}\vartheta_{ri}^{2}\left(k\right)\\ \; & +2\delta_{rim}^{2}+2Y_{1m}^{2}\\ \; & -\beta^{k}Q_{i}\left(k\right)-\varrho^{k}R_{i}\left(k\right) \end{array}$$

If the conditions are met, then we can obtain

$$\begin{array}{cc} \; & \alpha_{ri}\leq \frac{1}{\left|\right|\Delta_{ri}\left(k\right){\left|\right|}^{2}},\alpha_{ci}\leq \frac{1}{\beta^{2}\left|\right|\Delta_{ci}\left(k\right){\left|\right|}^{2}},\alpha_{ai}\leq \frac{1}{\left|\right|\Delta_{ai}\left(k\right){\left|\right|}^{2}}\\ \; & \left|\right|\delta_{ci}\left(k\right)\left|\right|>\sqrt{\left(V_{1m}^{2}+2W_{1m}^{2}X_{1m}^{2}+4\omega_{a2im}^{2}\Delta_{aim}^{2}+2\delta_{rim}^{2}+2Y_{1m}^{2}\right)/\beta^{2}} \end{array}$$

We can derive ΔL(k)≤0. The proof has been completed.

(2) In the absence of the triggering conditions, consider the following:

(61) $$L\left(k\right)=L_{1}\left(k\right)+L_{2}\left(k\right)+L_{4}\left(k\right)$$

where

$$\begin{array}{cc} \; & L_{1}\left(k\right)=\frac{1}{\alpha_{ri}}tr\left(\omega_{r2i}^{T}\left(k\right)\omega_{r2i}\left(k\right)\right),\\ \; & L_{2}\left(k\right)=\frac{1}{\alpha_{ci}}tr\left(\omega_{c2i}^{T}\left(k\right)\omega_{c2i}\left(k\right)\right),\\ \; & L_{4}\left(k\right)=e_{i}^{T}\left(k\right)e_{i}\left(k\right) \end{array}$$

(62) $$\begin{array}{cc} \Delta L(k) & =\Delta L_{1}\left(k\right)+\Delta L_{2}\left(k\right)+\Delta L_{4}\left(k\right)\\ \; & =-\beta^{2}\left|\right|\delta_{ci}{\left(k\right)||}^{2}-\beta^{2}(1-\alpha_{ci}\beta^{2}\left|\right|\Delta_{ci}{\left(k\right)||}^{2})\\ \; & \times \left|\right|\delta_{ci}\left(k\right)+\beta^{-1}V_{1}{\left(k\right)||}^{2}+\left|\right|V_{1}\left(k\right){\left|\right|}^{2}\\ \; & -(1-\alpha_{ri}\left|\right|\Delta_{ri}{\left(k\right)||}^{2})\varrho^{2}\left|\right|Y_{1}\left(k\right){\left|\right|}^{2}\vartheta_{ri}^{2}\left(k\right)\\ \; & +2\delta_{rim}^{2}+2Y_{1m}^{2}\\ \; & +e_{i}^{T}\left(k+1\right)e_{i}\left(k+1\right)-e_{i}^{T}\left(k\right)e_{i}\left(k\right) \end{array}$$

(63) $$\begin{array}{cc} \Delta L(k) & \leq -\beta^{2}\left|\right|\delta_{ci}{\left(k\right)||}^{2}-\beta^{2}(1-\alpha_{ci}\beta^{2}\left|\right|\Delta_{ci}{\left(k\right)||}^{2})\\ \; & \times \left|\right|\delta_{ci}\left(k\right)+\beta^{-1}V_{1}{\left(k\right)||}^{2}+\left|\right|V_{1}\left(k\right){\left|\right|}^{2}\\ \; & -(1-\alpha_{ri}\left|\right|\Delta_{ri}{\left(k\right)||}^{2})\varrho^{2}\left|\right|Y_{1}\left(k\right){\left|\right|}^{2}\vartheta_{ri}^{2}\left(k\right)\\ \; & +2\delta_{rim}^{2}+2Y_{1m}^{2}\\ \; & +\left((\iota |\right|e_{i}\left(k\right)+\iota ||\epsilon_{i}^{s}{\left||\right)}^{2}-\left|\right|e_{i}{\left(k\right)||}^{2})\\ \; & \leq -\beta^{2}\left|\right|\delta_{ci}{\left(k\right)||}^{2}-\beta^{2}(1-\alpha_{ci}\beta^{2}\left|\right|\Delta_{ci}{\left(k\right)||}^{2})\\ \; & \times \left|\right|\delta_{ci}\left(k\right)+\beta^{-1}V_{1}\left(k\right){\left|\right|}^{2}+V_{1m}^{2}\\ \; & -(1-\alpha_{ri}\left|\right|\Delta_{ri}{\left(k\right)||}^{2})\varrho^{2}\left|\right|Y_{1}\left(k\right){\left|\right|}^{2}\vartheta_{ri}^{2}\left(k\right)\\ \; & +2\delta_{rim}^{2}+2Y_{1m}^{2}\\ \; & -\left(1-2\iota^{2}\right)\left|\right|e_{i}\left(k\right){\left|\right|}^{2}-2\iota^{2}\left|\right|\epsilon_{i}^{s}{\left|\right|}^{2} \end{array}$$

In the event that it is satisfied that $\alpha_{ri}\leq \frac{1}{\left|\right|\Delta_{ri}\left(k\right){\left|\right|}^{2}},\alpha_{ci}\leq \frac{1}{\beta^{2}\left|\right|\Delta_{ci}\left(k\right){\left|\right|}^{2}},\alpha_{ai}\leq \frac{1}{\left|\right|\Delta_{ai}\left(k\right){\left|\right|}^{2}}$, and $\left|\right|\delta_{ci}\left(k\right)\left|\right|>\sqrt{\left(V_{1m}^{2}+2\delta_{rim}^{2}+2Y_{1m}^{2}\right)/\beta^{2}}$, one has ΔL(k)≤0. Thus, we can derive ΔL(k)≤0, and the proof is completed.

## 6. Statistical Data Illustration

To demonstrate the viability of the proposed method, a simulation is presented in the following section.

### Nonlinear MAS Consisting of One Leader and Six Followers

**Figure 1:**
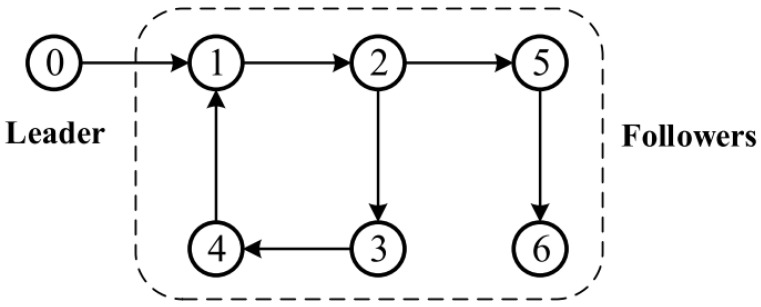
The topology structure for leader-follower MASs.

There were six followers and one leader in this tangled set of MASs which were considered. Figure 1 depicts the connection graph of the studied MASs. There was a leader of 0, and there were followers of 1, 2, 3, 4, 5, and 6. It is possible to obtain the corresponding adjacency matrix $a_{14}=a_{21}=a_{32}=a_{43}=a_{52}=a_{65}=1$. There is a weighted relationship involving the leaders and followers where $b_{1}=1,b_{2}=b_{3}=b_{4}=b_{5}=b_{6}=0$. It is possible for agent 1 to accept the information of the leader immediately. The system model parameters for MASs with one leader as well as six followers are as follows: $A=\left|\begin{array}{cc} 0.995 & 0.09980\\ -0.09982 & 0.995 \end{array}\right|,\;B_{1}={\left[0,0.2\right]}^{T},\;B_{2}={\left[0,0.5\right]}^{T},\;B_{3}={\left[0,0.4\right]}^{T},\;B_{4}={\left[0,0.3\right]}^{T},\;B_{5}={\left[0,0.6\right]}^{T}$, and $B_{6}={\left[0,0.7\right]}^{T}$.

The weight matrices are as follows: $Q_{11}=Q_{22}=Q_{33}=Q_{44}=Q_{55}=Q_{66}=1,R_{11}=R_{22}=R_{33}=R_{44}=R_{55}=R_{66}=I_{2\times 2}$, and $Q_{14}=Q_{21}=Q_{32}=Q_{43}=Q_{52}=Q_{65}=I_{2\times 2}$. The learning rates are $\alpha_{ri}=0.95,\alpha_{ai}=0.90$, and $\alpha_{ci}=0.07\left(iisequalto1,2,3,4,5,6\right)$, with a discount factor of ϱ=0.57,β=0.9.

For the agents, the activation function of the RNNs and ANNs is as follows: $Z_{r1}\left(k\right)={\left[e_{1}^{T}\left(k\right),u_{1}^{T}\left(kt_{s}^{1}\right),u_{4}^{T}\left(kt_{s}^{4}\right)\right]}^{T},$$Z_{a1}\left(k\right)=e_{1}\left(kt_{s}^{1}\right),$$Z_{r2}\left(k\right)={\left[e_{2}^{T}\left(k\right),u_{2}^{T}\left(kt_{s}^{2}\right),u_{1}^{T}\left(kt_{s}^{1}\right)\right]}^{T},$$Z_{a2}\left(k\right)=e_{2}\left(kt_{s}^{2}\right),$$Z_{r3}\left(k\right)={\left[e_{3}^{T}\left(k\right),u_{3}^{T}\left(kt_{s}^{3}\right),u_{2}^{T}\left(kt_{s}^{2}\right)\right]}^{T},$$Z_{a3}\left(k\right)=e_{3}\left(kt_{s}^{3}\right),$$Z_{r4}\left(k\right)={\left[e_{4}^{T}\left(k\right),u_{4}^{T}\left(kt_{s}^{4}\right),u_{3}^{T}\left(kt_{s}^{3}\right)\right]}^{T},$

$Z_{a4}\left(k\right)=e_{4}\left(kt_{s}^{4}\right),$$Z_{r5}\left(k\right)={\left[e_{5}^{T}\left(k\right),u_{5}^{T}\left(kt_{s}^{5}\right),u_{2}^{T}\left(kt_{s}^{2}\right)\right]}^{T},$$Z_{a5}\left(k\right)=e_{5}\left(kt_{s}^{5}\right),$$Z_{r6}\left(k\right)={\left[e_{6}^{T}\left(k\right),u_{6}^{T}\left(kt_{s}^{6}\right),u_{5}^{T}\left(kt_{s}^{5}\right)\right]}^{T},$$Z_{a6}\left(k\right)=e_{6}\left(kt_{s}^{6}\right).$ The initial values of the leader and followers are $x_{0}\left(0\right)={\left[0.6675,0.7940\right]}^{T},x_{1}\left(0\right)={\left[0.5734,0.6000\right]}^{T},x_{2}\left(0\right)={\left[0.5667,0.7348\right]}^{T},x_{3}\left(0\right)={\left[0.8694,0.7140\right]}^{T},x_{4}\left(0\right)={\left[1.0212,1.3842\right]}^{T},x_{5}\left(0\right)={\left[0.8606,1.5565\right]}^{T}$, and $x_{6}\left(0\right)={\left[0.5274,1.3235\right]}^{T}$.

**Figure 2:**
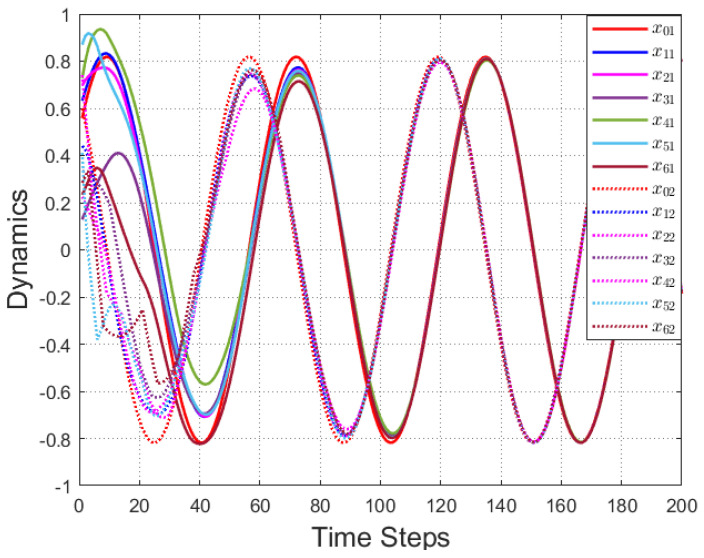
The tracks for the leader and followers.

**Figure 3:**
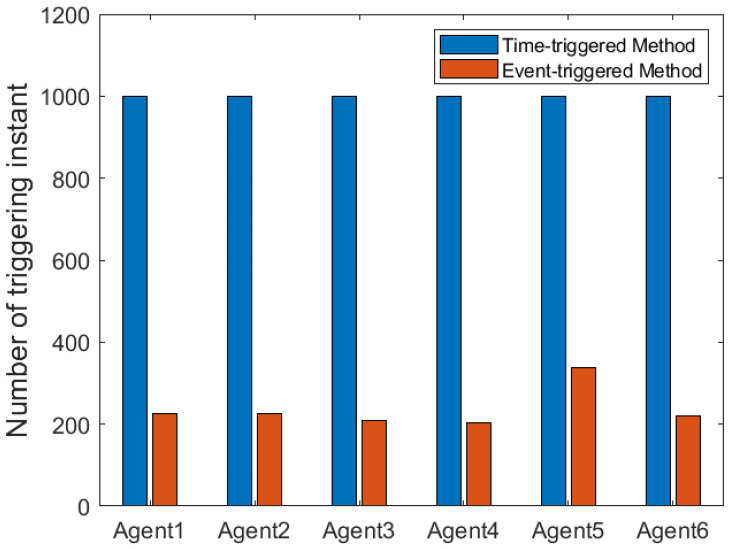
The comparison of the trigger time number involving the suggested method as well as the conventional approach.

**Figure 4:**
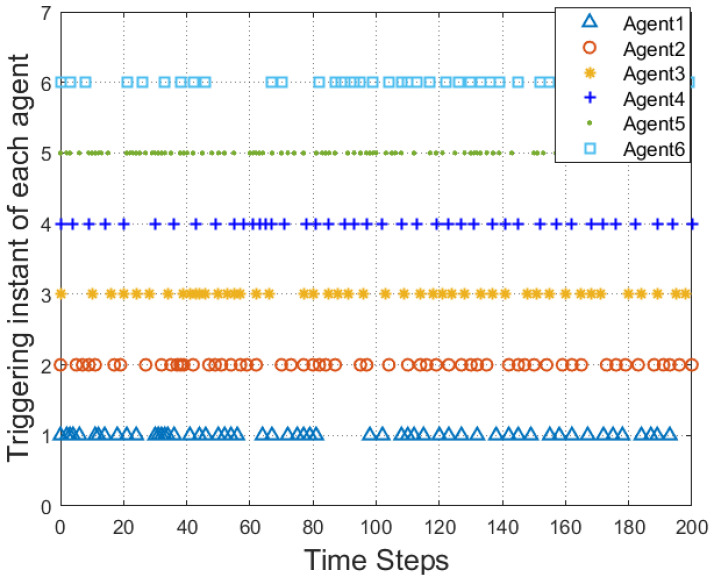
The triggering instant for each agent.

**Figure 5:**
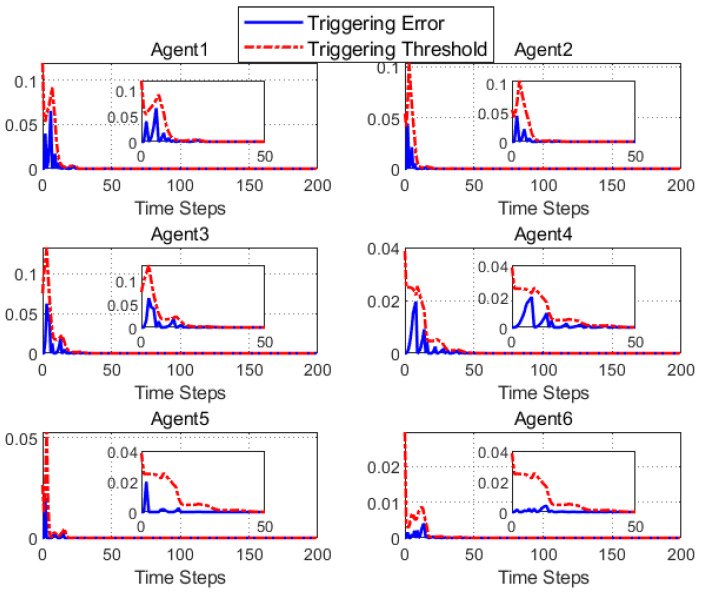
The triggering error trajectory $\left|\right|\epsilon_{i}^{s}\left(k\right){\left|\right|}^{2}$ in addition to triggering thresholds $\pi_{i}T\left(i=1,2,3,4,5,6\right)$.

**Figure 6:**
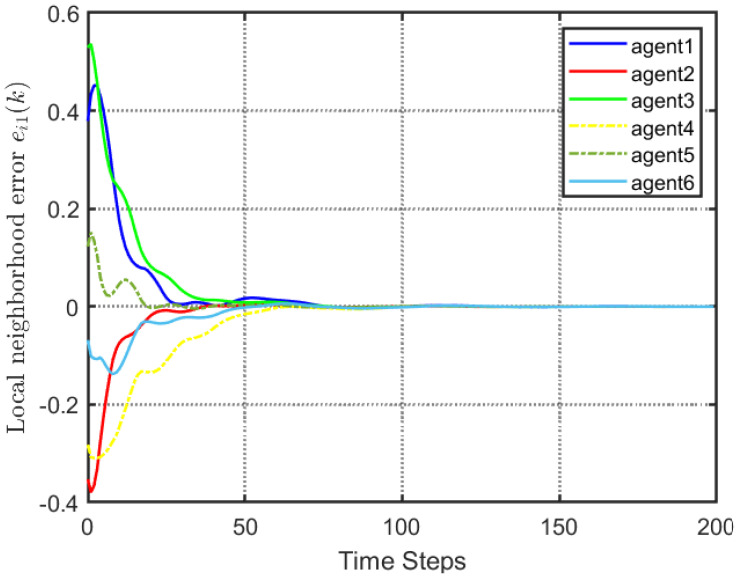
Local neighborhood errors $e_{i1}\left(k\right)$ with the proposed control method.

**Figure 7:**
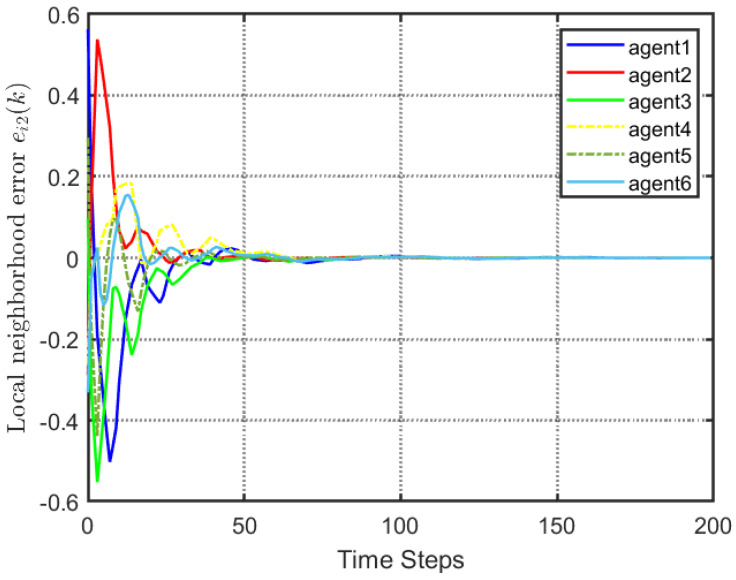
Local neighborhood errors $e_{i2}\left(k\right)$ with proposed control method.

**Figure 8:**
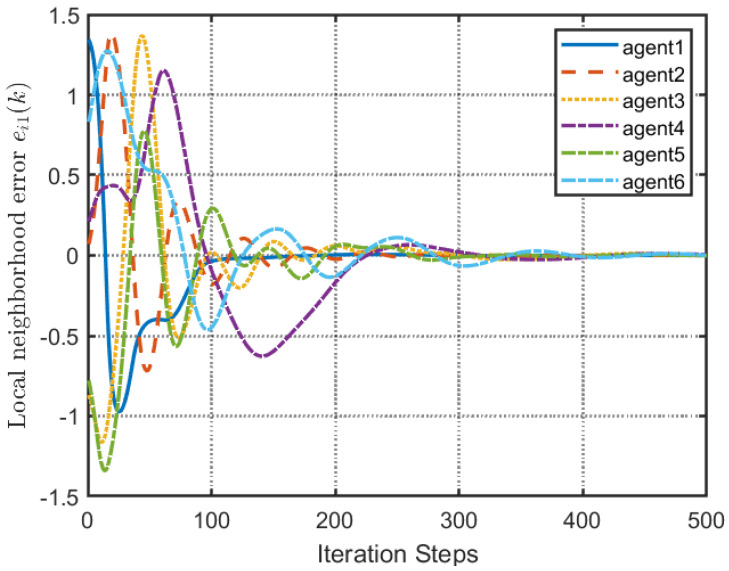
Local neighborhood errors $e_{i1}\left(k\right)$ of [32].

**Figure 9:**
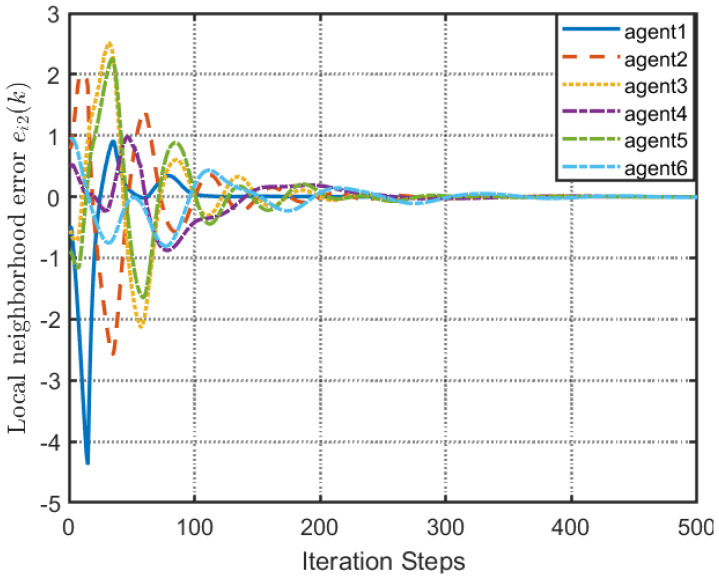
Local neighborhood errors $e_{i2}\left(k\right)$ of [32].

**Figure 10:**
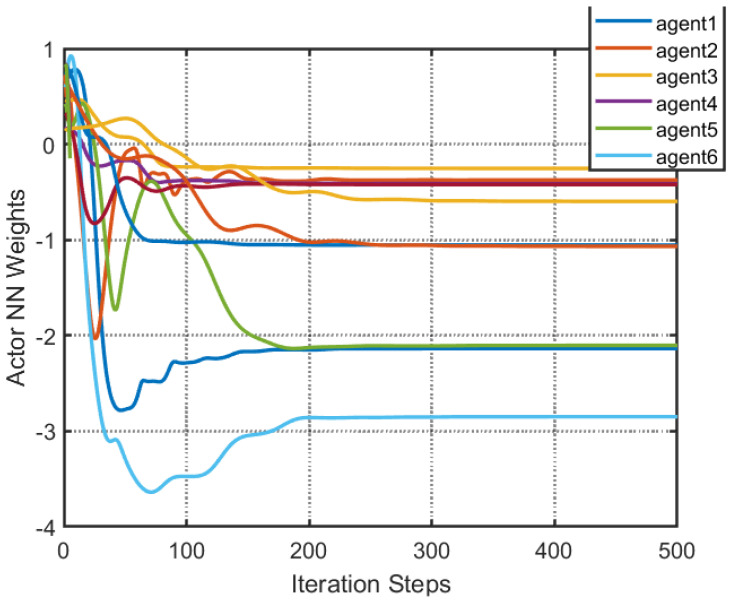
The estimation of weight parameters of the ANN of [32].

**Figure 11:**
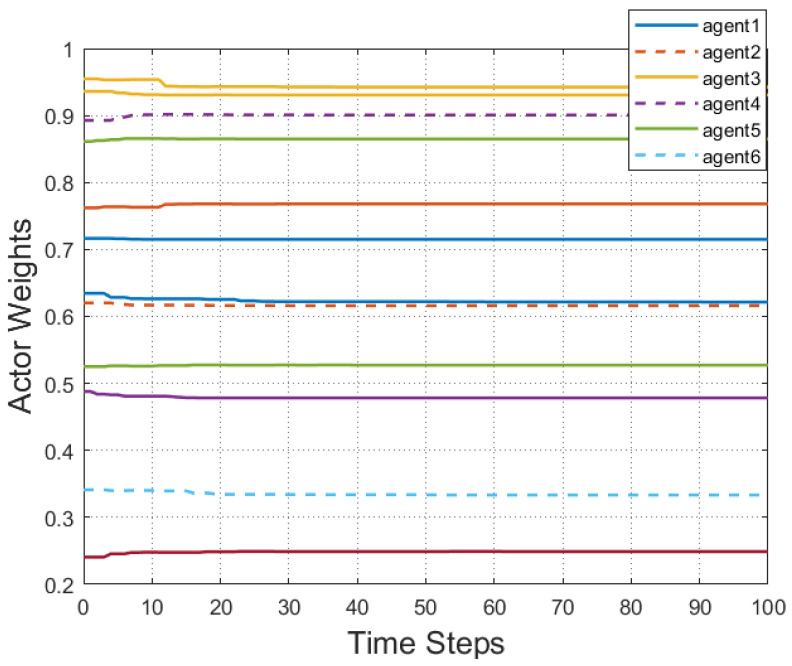
Estimation of the weight parameters of an ANN using the proposed control method.

According to Figure 2, all followers of the leader were able to accurately follow the leader, and the whole MAS was able to achieve synchronization. Figure 3 illustrates the six agents’ cumulative amount of trigger instants. On average, the amount of trigger instants for the six agents was approximately 220. However, using the traditional RL method, the number was approximately 1000. As a result, the computational burden was reduced by 78.0% in comparison with the conventional time-triggered method. According to Figure 4, the trigger mechanism of each agent is illustrated, which indicates that the actor network weight will be updated only when the trigger mechanism is satisfied. As can be seen in Figure 5, there is a correlation involving the error of triggering $\left|\right|\epsilon_{i}^{s}\left(k\right){\left|\right|}^{2}$ as well as the minimum triggering requirements $\pi_{i}T$. Over time, it appears that the triggering error converged. Figure 6 and Figure 7 illustrate the evaluation of the local neighborhood errors using the proposed control method, and it is shown that they could be converged to 0 at *k* = 60. The local neighborhood errors of [32] are shown in Figure 8 and Figure 9. In comparison with Figure 8 and Figure 9, our proposed control method produced a better convergence effect. Figure 10 and Figure 11 show the estimation of the ANN weight parameters. With the proposed control method, the actor network weights can stabilize faster than with IrQL.

## 7. Conclusions

According to this study, an event-triggered optimum controlling problem for model-free MASs was examined using the IrQL method based on RL. A new IrQL method was introduced by adding additional IRR functions [32], As a result, more information could be obtained by the agent. As a consequence of defining the IRR formula, we defined the Q-function and derived the corresponding HJB equation. In an iterative approach to IrQL, this method was designed to calculate the optimal control strategy. Using the IrQL algorithm, an event-triggered controller utilizing the IrQL method was presented. It was designed to update the controller only at the time of triggering to reduce the burden on computing resources and the transmission network. An RCA-NN was used to implement the suggested approach, which eliminated the need for a model of the system. It is possible to determine the convergent weights of neural networks using the Lyapunov method. To assess the performance and control efficiency of the suggested algorithm, a simulation model was used. Further research will be conducted on the effect of the discount rates on system reliability.
